# Prevalence of Diabetes and Prediabetes among Children Aged 11-14 Years Old in Vietnam

**DOI:** 10.1155/2020/7573491

**Published:** 2020-03-01

**Authors:** Duong H. Phan, Vuong V. Do, Long Q. Khuong, Hung T. Nguyen, Hoang V. Minh

**Affiliations:** ^1^National Hospital of Endocrinology, Hanoi, Vietnam; ^2^Center for Population Health Sciences, Hanoi University of Public Health, Hanoi, Vietnam; ^3^National Institute of Nutrition, Hanoi, Vietnam

## Abstract

**Aim:**

Diabetes in children is becoming more prevalent in some countries. However, in most countries, little is known about the epidemiology of this disease. This study is aimed at estimating the prevalence of type 1 and type 2 diabetes and prediabetes among children in Vietnam and examining factors associated with the conditions.

**Methods:**

A total of 2880 students aged 11-14 years old were recruited for the survey, using a school-based and nationally representative sampling frame. Capillary blood samples of participants were collected to measure fasting glucose level, using glucose meter OneTouch Verio Pro+. Diabetes and impaired fasting plasma glucose were initially diagnosed based on the cut-off points of the American Diabetes Association criteria. Diabetes status and type of diabetes of participants were confirmed at a hospital. Additionally, anthropometric and blood pressure measurements were conducted following a standardized procedure. Multivariate logistic regression was used to examine the association between outcome and independent variables.

**Results:**

The overall prevalence of diabetes among the participants was 1.04‰ (three cases), with 2 cases (0.75‰) diagnosed with type 1 diabetes (one known and one newly diagnosed) and 1 case newly diagnosed with type 2 diabetes (0.35‰). The prevalence of impaired fasting glucose was 6.1%. Body mass index, place of residence, and age were found to be significantly associated with the impaired fasting glucose condition in participants.

**Conclusion:**

The prevalence of type 1 and type 2 diabetes in children in Vietnam is lower than that in some other countries reported recently. However, there is a high prevalence in impaired fasting glucose, requiring attention from policymakers to take action to prevent the occurrence of the epidemic of type 2 diabetes in children in the future.

## 1. Introduction

The epidemic of diabetes is one of the major concerns for public health globally, and it is projected that 700 million adults aged 20-79 (10.9% of the population) will have diabetes by 2045 [1]. Type 2 diabetes (T2D) is the most prevalent form of diabetes and has increased alongside cultural and societal changes [1]. Not only highly prevalent in adults, T2D has also been increasing in youth [2]. In the USA, the incidence rates of T2D increased by 7.1% annually among youth aged 10-19, from 9.0 cases per 100,000 per year in 2002–2003 to 12.5 cases per 100,000 per year in 2011–2012 [3]. In some countries in Asia, such as Japan, the incidence rate of T2D among children aged 13-15 doubled from 7.3 per 100,000 between 1976 and 1980 to 13.9 per 100,000 in 1991-1995, and new T2D cases have dominated type 1 diabetes [4]. In Taiwan from 1992 to 1999, the incidence of newly diagnosed T2D among children aged 6-18 was 6.5 cases per 100,000, compared to 1.5 cases per 100,000 for type 1 [5]. Although an increasing trend is observed in some countries, population-based data about T2D in children are sparse and indeed absent in most countries.

The early onset of T2D in children makes lifetime exposure to hyperglycemia longer and consequently causes a greater risk for long-term complications [6]. In addition, the development of T2D in young people could be faster and more disruptive than in people with a later onset of the disease, causing early morbidity and reduced quality of life [7]. In comparison to type 1 diabetes, children with T2D are prone to a significantly higher risk of developing earlier and severe microvascular and cardiovascular diseases [8–10]. Some risk factors for developing childhood T2D are similar to those for adulthood, such as obesity, family history, and ethnicity [11]. Additionally, metabolic risk factors for T2D, including high blood pressure, high cholesterol, impaired glucose tolerance, and metabolic syndrome, are also associated with obesity [12, 13].

Vietnam is a developing country in Southeast Asia. The country has been undergoing a rapid epidemiological transition, with the emergence of noncommunicable disease as a critical public health issue, especially T2D. The prevalence of T2D doubled from 2.7% in 2002 to 5.4% in 2012 [14]. According to the International Diabetes Federation, the estimated number of Vietnamese people aged 20-79 with diabetes in 2019 was 3,779,600 cases [1]. T2D has been studied in adult populations; nevertheless, no study has been found to estimate the prevalence of diabetes among children in Vietnam. The prevalence of overweight and obesity among children in Vietnam is increasing, especially in urban areas. A study found that the prevalences of overweight and obesity among children aged 11-14 in a large city were 17.8% and 3.2%, respectively [15]. Therefore, this study is aimed at estimating the prevalence of type 1 and type 2 diabetes and prediabetes among Vietnamese children aged 11-14 and examining associated factors of the condition. Results of the study would add more epidemiological information about diabetes and prediabetes in children in developing countries as well as inform public health interventions in Vietnam.

## 2. Materials and Methods

### 2.1. Study Design and Population

A cross-sectional study was designed to estimate the prevalence of diabetes and prediabetes among children in Vietnam, with a school-based and nationally representative sampling frame. The National Hospital of Endocrinology of Vietnam led the study, and data was collected from September to November 2018. The probability proportional to size method and the sampling frame of the National Population and Housing census in 2009 [16] were used to randomly select thirty clusters (commune/ward) from each of the three regions of Vietnam (North, Center, and South), yielding a total of ninety selected clusters nationwide. Children who were studying at secondary school (grade six to nine) of the selected clusters were recruited for the study. In case a cluster has more than one secondary school, the school with the highest number of students was selected, whereas the nearest commune/ward replaced clusters without any secondary school. In each selected school, one class was randomly chosen to represent each grade. Eventually, in each selected class, eight students were randomly selected (four female students and four male students), making up a list of 32 students in each school and a total of 2880 students for the survey. Participants of the study were students who met the following criteria: (1) aged from 11 to 14 years old at the time of survey; (2) had no physical or mental illness; (3) willing to participate in the study; and (4) able to provide signed consent form by their parent or legal guardian. Students who did not meet the inclusion criteria or who wished to withdraw from the study at any stage were replaced by the next student in the class roster.

### 2.2. Data Collection Procedures

A study participation invitation letter, consent form, and self-administered questionnaire for parents were sent to the family of all selected children several days before the survey date. Data was collected in the mornings at the selected schools. In the documents delivered to families, students were told to have overnight fasting and skip breakfast (or have at least 8 hours of fasting) on the day of appointment for capillary blood collection. Students who came to the appointment were first screened by interviewers to check if they had met all inclusion criteria and their fasting status before proceeding to other steps of the survey, including anthropometric and blood pressure measurements, answering survey questionnaires, and testing blood glucose. All anthropometric and blood pressure measurements were performed according to a written standardized protocol.

Data collectors were trained researchers who have both a medical background and experience in conducting surveys. All these researchers participated in standard training conducted by the National Hospital of Endocrinology to make sure data was collected properly according to the protocol. The study's protocol was approved by the Committee on Human Research Ethics, while informed verbal and written consent was obtained from both students and their parents.

### 2.3. Measurements

The researchers collected a capillary blood sample of students. They then used the glucose meter OneTouch Verio Pro+ (LifeScan, Inc.) to measure fasting capillary blood glucose (FCG), which was used for the diagnosis of diabetes and prediabetes. FCG test can be an appropriate tool for mass screening to detect diabetes and prediabetes with acceptable test properties [17]. This study used the cut-off points for the diagnosis of prediabetes and diabetes based on the American Diabetes Association criteria [18]. In particular, impaired fasting glucose (IFG) was diagnosed if FCG levels fall between 100-125 mg/dl and 5.6-6.9 mmol/l, while diabetes was initially defined by FCG levels ≥ 126 mg/dl or 7.0 mmol/l or self-reported by the student. Students who had a FCG test diagnosed with diabetes were referred to a national or provincial hospital of the province/city where they were subjected to other tests and assessed by a physician to confirm the status of diabetes (type 1 or type 2 diabetes). Fasting plasma glucose and/or 2 h plasma glucose tests were performed to confirm diabetes status at the hospital. The type of diabetes was confirmed by further examinations of the physician (checking the presence of clinical risk factors, first-degree family history of diabetes) and the result of autoantibody tests.

The height of students was measured in centimeters (cm) by the Microtoise stature meter with the precision of 0.1 cm, while weight was measured in kilograms (kg) using a Tanita digital scale. Body mass index (BMI) was then calculated, and overweight and obesity were classified based on the gender- and age-specific BMI and World Health Organization (WHO) 2007 *Z*-score reference recommendations for children 5-19 years old [19]. Overweight was defined as a BMI value over +1 standard deviation (SD), while obesity as a BMI value over +2 SD of the gender- and age-specific reference population [19].

Systolic and diastolic blood pressure was measured by the ALPK 2 Aneroid sphygmomanometer (Tanaka Sangyo Co., Ltd, Japan). Hypertension was defined as systolic or diastolic blood pressure over 95^th^ percentile blood pressure specified for age, sex, and height of reference population [20]. Other variables, such as demographic information of students, history of diabetes, physical activity, and sedentary habits (watching TV/playing video game), were collected through an interviewer-administered questionnaire, whereas parents' history of diabetes and gestational diabetes was collected through a self-administered questionnaire sent to students' parents.

### 2.4. Statistical Analyses

Descriptive statistics were used to summarize the data, with frequencies and percentages for categorical variables and means and standard deviations for quantitative variables. Characteristics of participants related to diabetes and prediabetes were compared between groups using chi-square or Fisher exact tests for categorical variables. Odds ratio (OR) and 95% confidence intervals for the cross-tabulations were calculated using logistic regression. Multivariable logistic regression was conducted to identify factors related to prediabetes. Variables with a *p* value less than 0.2 in bivariate analyses were entered in the initial multivariable logistic regression model based on procedures recommended by Hosmer et al. [21]. Those variables, nonsignificant variables, from the initial multivariable model were dropped. The likelihood ratio test was used to compare the final model to the model with each nonsignificant variable included. A significant level of *p* < 0.05 was used for all statistical tests. All the statistical analyses were performed using R 3.5.0.

## 3. Results

Among the initial sample of 2880 students, 85 cases (3%) either did not wish to participate or were excluded from the study due to some reasons, including inability to provide signed parental consent form and not feeling physically well at the time of the survey. These 85 cases were replaced by their classmates, keeping our actual sample of study at 2880 students.

Due to the sampling technique, the mean age of the three regions was similar (12.4 ± 1.1 for students in the north and 12.5 ± 1.1 for students in the south and central region).  Table 1 provides information about characteristics of participants by regions. On average, nearly three out of five participants were living in rural areas, compared to two out of five in urban areas. The mean weight of participants was approximate 45 kg, and the mean height was 152 cm. Nearly 26% of students in the south drunk carbonated soft drinks for at least three times per week, whereas these proportions among students in north and central regions were about 18% and 23%, respectively. Only 9.1% of students in the south spent 60 minutes or more per day playing sports, compared to 13.3% of students in the central region and 15.6% of students in the north. In addition, about 19% of students in the south spent more than 3 hours watching TV/playing video games per day while the corresponding proportions of students in the central and northern regions were 5.8% and 10.4%, respectively.

Table 2 shows the prevalence of diabetes and IFG among participants in our study. There were three cases (1‰) of diabetes in total, with one known type 1 diabetes, and two newly identified diabetes cases. The two newly identified diabetes cases were further examined by physicians and were categorized as 1 new type 2 diabetes (0.35‰) and one new type 1 diabetes (0.35‰). One hundred seventy-five students (6.1%) were diagnosed with the IFG condition. There was no significant difference in the prevalence of prediabetes between the three regions of Vietnam and between male and female students. However, the prevalence of prediabetes differed between age groups, with the youngest age group (11 years old) having the highest percentage of prediabetes 8.1% (95% CI: 6.4-10.3), whereas other older age groups have a lower percentage of prediabetes.

The distribution of T2D risk factors by IFG status and among all participants was presented in Table 3. The proportion of obesity and overweight in study participants were 8.3% and 17.6%, respectively. The proportion of obese students among those who diagnosed with IFG almost doubled among those without IFG (14.9% vs. 7.9%). Hypertension was diagnosed in 5.2% of the total participants. In addition, a family history of diabetes was reported in 2.1%, while exposure to diabetes in utero was reported in only 0.4% of students. There was no statistically significant difference in the proportion of hypertension, family history of diabetes, and exposure to diabetes in utero between students with a diagnosis of IFG and those without IFG condition. In sum, 12.4% of students were found to have at least one T2D risk factor, and 1.8% have two or more T2D risk factors.

Factors associated with prediabetes among students were reported in Table 4. Both bivariate and multivariate analyses show that age, place of residence, and BMI were associated with prediabetes among students, whereas sex, ethnicity, region, hypertension status, family history of T2D, exposure to diabetes in utero, soft drink consumption, physical activity, and sedentary habits were not associated with prediabetes. Students who lived in urban areas were less likely to be diagnosed with prediabetes compared to those in rural areas (adjusted OR = 0.66, 95% CI: 0.48-0.92). Additionally, obese students were 2.1 times more likely to be diagnosed with prediabetes in comparison with normal weight students.

## 4. Discussion

This present study is the first study investigating the prevalence of diabetes and prediabetes among children ages 11-14 years old in Vietnam. The results showed that the prevalence of diabetes was 1.4‰. Noticeably, two-thirds of diabetes cases were newly identified, with half of the cases type 1 and half type 2 diabetes. The proportion of children diagnosed with the IFG condition was 6.1%, with no significant difference between groups across regions and gender. In addition, we found that BMI, place of residence, and age were significant predictors of prediabetes among Vietnamese children.

The prevalence of type 1 diabetes in our participants was 0.7‰. Due to the design of the study, we could not estimate the number of incident cases of type 1 diabetes for children in Vietnam. Globally, the estimated number of incident cases of type 1 diabetes was highest in India, with 15,900 cases, whereas the estimated incident cases of type 1 diabetes for Thailand, a country in Southeast Asia, were only 100 cases [22]. Given that the number of children and adolescents with type 1 diabetes worldwide continues to increase, follow-up studies are needed to estimate the incidence rate as well as monitor the trend of type 1 diabetes in children in Vietnam.

One-third of diabetes cases were T2D diabetes in our study, which is consistent with the increasing prevalence of T2D among children observed in some countries in the last decade [2]. The prevalence of T2D in our study was much lower than the prevalence among Brazilian school children aged 12-17 years old in 2013-2014 (3.3%) [23] and slightly lower than the prevalence reported in the USA in 2009 among children aged 10-19 years old (0.046%) [24]. Another school-based study in the Tokyo Metropolitan area of Japan reported the overall incidence of T2D (per 100,000 person-year) during the 1975-2015 period was 0.8 in students aged 6-12 years and 6.41 in students aged 13-15 years [25]. The incidence and prevalence of T2D vary substantially among countries due to not only differences in population characteristics but also methodological differences of studies, such as calendar time and duration of the study, study design and case ascertainment methods, and diagnosis of T2D and classification [5].

The prevalence of prediabetes depends on the conditions used for screening (impaired fasting glucose or impaired glucose tolerance) and the use of definitions. Impaired fasting glucose is defined as above 5.6 mmol/l by the American Diabetes Association [18] and above 6.1 by the World Health Organization [26]. It is challenging to compare and interpret the prevalence of prediabetes in children between countries due to several reasons, such as the difference in the definition used, age groups of the sample populations, or different settings (i.e., whole population vs. obese children). The prevalence of impaired fasting glucose among children found in our study (6.1%) was lower than the prevalence reported in the study in Brazil (22%) [23] though similar to the prevalence reported in the study in Saudi Arabia (6.12%) among children aged under 19 years old [27]. However, the prevalence of prediabetes in our study was double the prevalence among children and adolescent in urban South India in 2013 (3.4%) [28] and triple the prevalence reported in a Chinese study among children aged 6-17 (1.89%) [29].

The prevalence of prediabetes in children and adolescents is closely related to the increase in childhood obesity [30]. We also observed a higher prevalence of obese children with a diagnosis of IFG. In addition, urban children in our study were more likely to be diagnosed with IFG than rural children, which is consistent with the finding of the study in China [29]. Urban children may be associated with more unhealthy behaviors than their rural counterparts, such as higher consumption of fried foods and carbonated soft drinks, and higher prevalence of physical inactivity, which might be associated with the difference in IFG prevalence [29]. We found that the age of children was negatively associated with IFG; however, in our study, we only included participants aged 11-14 years old, so the association might not be significant for an expanded age group. The detection of prediabetes is highly dependent on the pubertal status of adolescents [31, 32], which was not examined in our study. Further studies are necessary to examine factors related to IFG among children in Vietnam.

Diabetes management in children is complicated due to unique aspects of this group, such as the ability to provide self-care, supervision in the childcare and school environment, and neurological vulnerability to hypoglycemia and hyperglycemia in young children, as well as possible adverse neurocognitive effects of diabetic ketoacidosis [33, 34]. Additionally, physicians and the health care system are not ready to handle the challenges associated with T2D in children [35]. To prevent the epidemic of T2D occurring in Vietnamese children, of which the financial and societal consequences are substantial, it is important to have a public response from now on. Prevention of T2D in children means the prevention of obesity as the effect of weight loss on comorbid conditions and the development of T2D has been proven [36, 37]. However, the prevention of obesity in children would not succeed without the recognition of the government, local communities, schools, and parents that this is a critical health problem [38]. Therefore, all the levels from family to the government in Vietnam need to pay more attention to the prevention and treatment of obesity in children and adolescents.

This study has some limitations. First, children aged 11-14 years old were sampled from schools. Therefore, those who did not attend schools were not included in the study, affecting the generalizability of the findings. However, the proportion of children at this age that do not attend schools in Vietnam is relatively small. Second, our estimate for the prevalence of T2D relied on one single new case, which might not be a reliable estimated nationwide prevalence. A larger sampling frame study is needed to validate the prevalence of T2D among children in Vietnam. Third, though capillary blood sampling is less invasive and appropriate for collecting blood in children, the accuracy of the test results is affected by blood sampling procedures and more subject to bias, compared to venous blood sampling to diagnose prediabetes. Besides, despite asking participants to fast overnight and skip breakfast before blood collection, we could not be sure that all children complied and truthfully answered screening questions.

## 5. Conclusions

This school-based study provides an estimate of the national prevalence of both type 1 and type 2 diabetes and prediabetes in children aged 11-14 years old in Vietnam. Despite the prevalence of type 1 and type 2 diabetes being lower than the prevalence reported in some countries recently, the prevalence of IFG in Vietnamese children is high, suggesting the need for public health interventions to prevent and decrease the rising prevalence of obesity in children.

## Figures and Tables

**Table 1:** Characteristics of participants.

Characteristics	North (*n* = 960)	Center (*n* = 960)	South (*n* = 960)	Total (*n* = 2880)
Age^a^				
11 years	262 (27.3)	253 (26.4)	251 (26.1)	766 (26.6)
12 years	243 (25.3)	239 (24.9)	236 (24.6)	718 (24.9)
13 years	228 (23.8)	252 (26.2)	258 (26.9)	738 (25.6)
14 years	227 (23.6)	216 (22.5)	215 (22.4)	658 (22.8)
Sex^a^				
Male	481 (50.1)	476 (49.6)	487 (50.7)	1444 (50.1)
Female	479 (49.9)	484 (50.4)	473 (49.3)	1436 (49.9)
Ethnicity^a^				
Kinh	735 (76.6)	836 (87.1)	848 (88.3)	2419 (84.0)
Others	225 (23.4)	124 (12.9)	112 (11.7)	461 (16.0)
Place of residence^a^				
Rural	672 (70.0)	608 (63.3)	416 (43.3)	1696 (58.9)
Urban	288 (30.0)	352 (36.7)	544 (56.7)	1184 (41.1)
Weight (kg)^b^	43.5 ± 10.6	43.2 ± 11.2	47.8 ± 13.2	44.8 ± 11.9
Height (cm)^b^	151.5 ± 9.4	151.8 ± 9.3	152.7 ± 9.3	152.0 ± 9.4
Waist circumference (cm)^b^	64.6 ± 8.3	63.1 ± 9.3	66.8 ± 10.7	64.8 ± 9.6
Hip circumference (cm)^b^	79.9 ± 8.5	75.4 ± 10.6	83.9 ± 10.0	79.7 ± 10.3
WTH ratio^b^	0.8 ± 0.1	0.8 ± 0.1	0.8 ± 0.1	0.8 ± 0.1
BMI (kg/m^2^)^b^	18.8 ± 3.3	18.5 ± 3.5	20.3 ± 4.4	19.2 ± 3.9
Systolic blood pressure (mmHg)^b^	100.6 ± 11.3	103.1 ± 11.5	94.1 ± 12.0	99.3 ± 12.2
Diastolic blood pressure (mmHg)^b^	63.5 ± 8.2	64.0 ± 8.5	59.3 ± 8.0	62.2 ± 8.5
Body fat percentage^b^	19.1 ± 9.2	19.3 ± 10.3	23.5 ± 12.1	20.7 ± 10.8
Drink carbonated soft drinks^a^				
No drink	482 (50.2)	357 (37.2)	247 (25.7)	1086 (37.7)
1-2 times/week	306 (31.9)	380 (39.6)	370 (38.5)	1056 (36.7)
≥3 times/week	172 (17.9)	223 (23.2)	343 (35.7)	738 (25.6)
Play any sports for at least 60 minutes per day^a^				
No	810 (84.4)	832 (86.7)	873 (90.9)	2515 (87.3)
Yes	150 (15.6)	128 (13.3)	87 (9.1)	365 (12.7)
Time watching TV/playing video game per day^a^				
<2 hours	457 (47.6)	612 (63.8)	398 (41.5)	1467 (50.9)
2–3 hours	402 (41.9)	292 (30.4)	382 (39.8)	1076 (37.4)
>3 hours	100 (10.4)	56 (5.8)	180 (18.8)	336 (11.7)

WTH: waist-to-hip ratio; BMI: body mass index. ^a^*n* (%). ^b^Mean ± SD.

**Table 2:** Prevalence of children with diabetes and impaired fasting glucose.

	Diabetes		Impaired fasting glucose		*p* ^∗^
	*n*	‰ (95% CI)	*n*	% (95% CI)	
Total	3	1.04 (0.2–3.2)	175	6.1 (5.3; 7.0)	
Region					
North	2	2.1 (0.05–8.1)	64	6.7 (5.2; 8.4)	0.086
Center	0	—	45	4.7 (3.5; 6.2)	
South	1	—	66	6.9 (5.4; 8.7)	
Sex					
Male	1	—	89	6.2 (5.0; 7.5)	0.845
Female	2	1.4 (0.03–5.4)	86	6.0 (4.9; 7.3)	
Age					
11	0	—	62	8.1 (6.4; 10.3)	0.022
12	3	4.1 (0.8–12.5)	46	6.4 (4.8; 8.5)	
13	0	—	36	4.9 (3.5; 6.7)	
14	0	—	31	4.7 (3.3; 6.6)	

^∗^ *p* value of tests to compare the prevalence of prediabetes between groups within region, sex, and age variables.

**Table 3:** Distribution of type 2 diabetes risk factors in total participants and impaired fasting glucose cases.

T2D risk factors	Total, *n* (%)	Impaired fasting glucose		*p*
		No (*n* = 2705)	Yes (*n* = 175)	
BMI, *n* (%)				
Normal	2134 (74.1)	2016 (74.5)	118 (67.4)	0.005
Overweight	506 (17.6)	475 (17.6)	31 (17.7)	
Obesity	240 (8.3)	214 (7.9)	26 (14.9)	
Hypertension^a^, *n* (%)				
No	2729 (94.8)	2564 (94.8)	165 (94.3)	0.910
Yes	151 (5.2)	141 (5.2)	10 (5.7)	
Family history of diabetes, *n* (%)				
No	2820 (97.9)	2648 (97.9)	172 (98.3)	1.000
Yes	60 (2.1)	57 (2.1)	3 (1.7)	
Exposure to diabetes in utero, *n* (%)				
No	2868 (99.6)	2693 (99.6)	175 (100.0)	1.000
Yes	12 (0.4)	12 (0.4)	0 (0.0)	
Number of risk factors^b^, *n* (%)				
0 risk factor	2471 (85.8)	2331 (86.2)	140 (80.0)	0.121
1 risk factor	356 (12.4)	325 (12.0)	31 (17.7)	
2 risk factors	52 (1.8)	48 (1.8)	4 (2.3)	
3 risk factors	1 (0.0)	1 (0.0)	0 (0.0)	

^a^Fisher exact test. ^b^Risk factors include obesity, hypertension, a family history of diabetes, exposure to diabetes in utero; none of students found to have all four risk factors.

**Table 4:** Factors related to impaired fasting glucose.

	OR	95% CI	*p*	aOR	95% CI	*p*
Age (year)	**0.81**	**0.70; 0.93**	**0.003**	**0.83**	**0.72; 0.95**	**0.008**
Sex (ref: male)						
Female	0.97	0.71; 1.32	0.845	—	—	—
Ethnicity (ref: Kinh)						
Others	0.74	0.47; 1.17	0.202	—	—	—
Region (ref: north)						
Middle	0.69	0.47; 1.02	0.062	—	—	—
South	1.03	0.72; 1.48	0.856	—	—	—
Place of residence (ref: rural)						
Urban	**0.70**	**0.50; 0.96**	**0.028**	**0.66**	**0.48; 0.92**	**0.014**
BMI (ref: normal)						
Overweight	1.12	0.74; 1.68	0.601	1.13	0.75; 1.71	0.558
Obesity	**2.08**	**1.33; 3.25**	**0.001**	**2.10**	**1.31; 1.71**	**0.002**
Hypertension (ref: no)						
Yes	1.10	0.57; 2.13	0.773	—	—	—
Family history of T2D (ref: no)						
Yes	0.81	0.25; 2.61	0.725	—	—	—
Exposure to diabetes in utero (ref: no)						
Yes	—	—	—	—	—	—
Drink carbonated soft drinks (ref: no drink)						
1–2 times/week	0.88	0.62; 1.25	0.473	—	—	—
≥3 times/week	0.77	0.52; 1.16	0.210	—	—	—
Play sport at least 60 minutes per day (ref: no)						
Yes	0.73	0.44; 1.22	0.226	—	—	—
Time watching TV/playing video game per day (ref: <2 hours)						
2–3 hours	0.88	0.64; 1.23	0.460	—	—	—
>3 hours	0.82	0.49; 1.37	0.446	—	—	—

Abbreviations: T2D: type 2 diabetes; OR: odds ratio; aOR: adjusted odds ratio; CI: confidence interval.

## Data Availability

The data used to support the findings of this study are available from the corresponding author upon reasonable request.
